# Genome-wide discovery and development of polymorphic microsatellites from *Leishmania panamensis* parasites circulating in central Panama

**DOI:** 10.1186/s13071-015-1153-2

**Published:** 2015-10-12

**Authors:** Carlos M. Restrepo, Alejandro Llanes, Carolina De La Guardia, Ricardo Lleonart

**Affiliations:** Instituto de Investigaciones Científicas y Servicios de Alta Tecnología (INDICASAT AIP), Building 219, Ciudad del Saber, Apartado 0843-01103, Ciudad de Panamá, República de Panamá; Department of Biotechnology, Acharya Nagarjuna University, Guntur, India

**Keywords:** *Leishmania panamensis*, Genome-wide screening, Microsatellite, Polymorphisms

## Abstract

**Background:**

The parasite *Leishmania panamensis* is the main cause of leishmaniasis in Panama. The disease is largely uncontrolled, with a rising incidence and no appropriate control measures. While microsatellites are considered some of the best genetic markers to study population genetics and molecular epidemiology in these and other parasites, none has been developed for *L. panamensis*.

**Findings:**

Here we have developed and tested a new panel of microsatellites for this species, based on high-throughput genome-wide screening. The new set of microsatellites is composed of seventeen loci, mainly spanning trinucleotide or longer motifs. We have evaluated the sensitivity and specificity of the panel based on a sample of 27 isolates obtained from cutaneous leishmaniasis patients from central Panama and also several reference species from both *L*. (*Leishmania*) and *L*. (*Viannia*) subgenera. The genetic equilibrium was assessed both intra- and inter-loci, while the reproductive mode was evaluated using several tests. The new SSR panel shows high polymorphism and sensitivity, as well as good specificity. The preliminary data described here for *L. panamensis* suggest extensive departure from Hardy-Weinberg proportions, significant linkage disequilibrium and strong deficit of heterozygotes. Several recombination tests involving multilocus linkage disequilibrium and a phylogenetic approach allowed rejection of frequent recombination in our dataset.

**Conclusions:**

The genome-wide strategy described here proved to be useful to identify and test new polymorphic SSR loci in *Leishmania*. The new panel of polymorphic microsatellites is a valuable contribution to the existing molecular markers for the study of genetic structure and other aspects of this important species.

**Electronic supplementary material:**

The online version of this article (doi:10.1186/s13071-015-1153-2) contains supplementary material, which is available to authorized users.

## Findings

In Panama, *Leishmania* (*Viannia*) *panamensis* is responsible for most of the reported clinical cases [1], causing cutaneous and mucocutaneous leishmaniasis. Some previous works have explored the genetic composition of local populations of this important parasite using kinetoplast DNA RFLP [2] and AFLP [3]. As these systems have some known limitations, more powerful genetic markers are needed for this species. Full genome sequencing is increasingly being used to uncover a vast number of DNA polymorphisms in many organisms; most of them being single nucleotide polymorphisms (SNPs). While genome-wide SNP genotyping is becoming more feasible and attractive for population genetics and molecular epidemiology in many organisms, markers such as microsatellites may still have advantages over SNPs due to high polymorphism information content and fast mutation rate [4]. Microsatellites (also simple sequence repeats, SSR) are still considered very useful markers for applications involving short temporal/spatial scales, or slowly evolving or clonal organisms [4–6].

There is growing evidence that some SSR may have low transferability among species, limiting the usefulness of these markers for interspecies studies [7, 8]. Additionally, the high intraspecific genetic variability reported for species of the *L*. (*Viannia*) subgenus further warrants the necessity to develop species-specific microsatellite panels. In this study we describe, develop and characterize a novel panel of microsatellite markers specific for *L. panamensis* based on genome-wide high-throughput screening (Additional file 1: Extended Methods). We have used for this purpose the sequenced *L. panamensis* chromosomes (Genbank accession numbers CP009370 to CP009404) [9]. Ethical approval for the experiments described here was obtained from INDICASAT AIP Institutional Review Board (Additional file 1: Extended Methods). As far as we know, this is the first microsatellite panel developed for *Leishmania panamensis*, using a bioinformatic pipeline at genomic level. We demonstrate that this new microsatellite panel is highly polymorphic and has good sensitivity and specificity, making it very promising for studying the genetic diversity in this parasite.

### *In silico* microsatellite screening and polymorphism assessment

The SSR mining procedure we used on the reference *L. panamensis* genome allowed the detection of a large number of potential loci, being the AC, AG, AGC, AT, ACC, AGG and GC the most frequent motifs (Additional file 1: Extended Methods; Additional file 2: Table S1). This distribution of abundance of SSR types differ from what was found in *L. braziliensis* [10]. However, results are not directly comparable as different search strategies were used. Out of 19,297 perfect microsatellite loci, 18,921 were considered appropriate for primer design and 12,871 flagged as potentially unique sequences. Four hundred and six microsatellite loci including di-, tri-, tetra- and pentanucleotides were evaluated for consistent amplification and polymorphism using two DNA pools, each one containing DNA from 10 different *L. panamensis* isolates. After several steps of selection, 104 potentially polymorphic loci were tested for polymorphism and consistent amplification patterns with DNA from individual isolates. All SSR loci were detected by fluorescent capillary electrophoresis using a three primer method (Additional file 1: Extended Methods). Finally, 17 microsatellite loci were selected, showing coherent segregation patterns of all peaks found in the pools, as well as low levels of stuttering (Table 1 and Table 2). The new set of *L. panamensis* microsatellites was validated by genotyping a group of 27 isolates obtained from cutaneous leishmaniasis patients from the central region of Panama (Additional file 1: Extended Methods).

**Table 1:** Primer sequences and locus characteristics.

Locus	Primers (5´ → 3´)^a^	Chr^b^	Loc^c^	Repeat motif	Region^d^	Ta^e^ (°C)	Fragment sizes (bp)^f^
M75	TCCCTAATCGTCCGTGTCAC	35	2041025	AGG	NC	56	258-270
	CAGTGACGAGCTGGAAATGC						
M78	AACAAGGCACGGGAAATTGG	35	2220491	AAC	NC	56	333-339
	CGTCTGTTGCTCCGTCTTTG						
M80	CCGCCGAGAAGCCTGAAG	29	193463	AGC	C	56	237-255
	GCAACAGTACGAGAAGTGGC						
M81	CATCATTCAGCAGTGCGGG	29	246586	AGG	NC	56	329-347
	AAAGGAAGTGATGCGTGCAC						
M140	GATTGTCGCCCTCATCAACG	17	494896	AGG	NC	64	236-263
	GAGAAGAGAGGGTCGAGCTC						
M147	TCTCCTTGGCTTTCCTCTCC	26	483774	AGG	NC	66	192-210
	ACATATTCGCGCGCCTAATG						
M149	TTCTGCCTTGTTGTTGCTCC	13	534713	AGG	NC	56	288-312
	CACATCGGCCTCCATAACAC						
M155	ACAAGAATGCCAAGCTGTCG	13	329823	AGC	C	62	314-323
	CCTCTGCTGCTTCCTGTTTC						
M166	ACTCCCTGGATAGTGGCAAC	7	107131	AGC	C	62	194-206
	ATGCGGTGCGTGATGTTATG						
M196	ATCAGCAGCAACTCTCCCAG	12	147953	AGC	C	62	288-300
	GAGATACCGGAGACAGCGG						
M202	CAGGGAGAAGAAGGGATGGC	5	267314	AGG	C	56	285-318
	TCCTTTGCCTCAGTCCGTAC						
M207	CTTTGACGGGCTCATCAGTG	5	251336	AGC	C	56	321-333
	AAGGAGCGAGAAGTAGAGGC						
M233	TCTGCGGTGAGGATGCTAC	3	102343	ACC	C	56	195-300
	GCTACTACCTCTGCTCACCC						
M269	TGCTGTTGACTGAATGCACTG	33	279723	AGG	NC	62	327-366
	TTTCCTCCACTCCCGTCTTC						
M292	GACTTAACGGATGCGCTGAC	20	158471	ACC	NC	62	240-252
	GGAGCACTGAAGTAAAGCGG						
M329	CCAGTCTCTTCCACCTACCC	27	715534	AGGG	NC	56	228-232
	GATGGAAGGGTGCACACATC						
M402	GCAGCAGTCGATTGTGAAGG	5	228009	AGAGG	NC	64	130-140
	CCCTTTACATGCTGCTTGGG						

^a^Upper line, forward primer; lower line, reverse primer. Forward primer had tail attached at 5´ (GCCTCCCTCGCGCC)

^b^Chromosomal position based on *L. panamensis* full genome assembly

^c^Chromosomal location, considering numbering of the first nucleotide of repeat 1

^d^Location of microsatellite in coding (C) or non-coding (NC) regions

^e^Annealing temperature

^f^Range of fragment sizes found in *L. panamensis* strains tested so far

**Table 2:** Summary of population genetics descriptive statistics of *L. panamensis* SSRs.

Locus	Repeat number^a^	Genotype no^b^	Na^c^	He^d^	Ho^e^	PIC^f^	*F*_IS_^g^	HWE^h^
M75	5-9	2	3	0.072	0.037	0.071	0.500	0.019
M78	6-8	3	3	0.171	0.185	0.161	−0.066	1.000
M80	2-8	3	3	0.172	0.111	0.165	0.371	0.022
M81	7-13	6	6	0.407	0.111	0.387	0.736	0.000
M140	6-15	9	7	0.682	0.185	0.639	0.737	0.000
M147	6-12	7	4	0.693	0.370	0.633	0.481	0.000
M149	5-13	5	5	0.444	0.556	0.409	−0.232	0.723
M155	7-10	4	3	0.612	0.111	0.532	0.825	0.000
M166	5-9	5	4	0.655	0.259	0.584	0.616	0.000
M196	3-7	3	3	0.535	0.000	0.426	1.000	0.000
M202	9-20	7	6	0.339	0.154	0.329	0.560	0.000
M207	7-11	4	3	0.536	0.077	0.469	0.861	0.000
M233	7-42	19	15	0.887	0.519	0.877	0.431	0.000
M269	9-22	5	6	0.385	0.111	0.372	0.720	0.000
M292	5-9	4	3	0.417	0.074	0.379	0.828	0.000
M329	5-6	3	2	0.431	0.111	0.338	0.751	0.000
M402	6-8	3	3	0.426	0.111	0.371	0.748	0.000
Overall		5.4	4.6	0.463	0.181	0.420	0.620	

^a^Number of repeats observed

^b^Number of different genotypes observed

^c^Number of alleles

^d^Expected heterozygosity

^e^Observed heterozygosity

^f^Polymorphism information content

^g^Wright inbreeding coefficient *F*_IS_

^h^ *P-values* of Hardy-Weinberg equilibrium exact test

We decided to exclude dinucleotide SSRs from the final panel, to improve allele assignment and stuttering levels. Additionally, we also chose to use perfect repeat loci to avoid errors due to ambiguous allele assignment and undefined marker evolution models [4].

None of the new microsatellite loci described showed more than two clear amplification peaks, suggestive of average diploidy in the corresponding chromosomes of the *L. panamensis* genome. This pattern of only 1–2 alleles per locus at microsatellite loci is consistently observed at each *Leishmania* species tested so far, including *L. panamensis* from Ecuador and Peru [11].

### Sensitivity, specificity and cross-species amplification of the microsatellite panel

Eleven loci could be amplified producing robust amplification signals even when tested with very low amounts of template *Leishmania* DNA (down to 0.01 ng) (Additional file 2: Table S3). These results may open the possibility to genotype parasites directly from infected tissues or insects with this new SSR panel. The described SSR set has high specificity for *Leishmania* DNA, showing no cross-reactivity with human DNA or with DNA from the insect vector, *Lutzomyia* sp. (Additional file 2: Table S2).

In general, the new microsatellite panel showed much better cross-species amplification with other species of the *L*. (*Viannia*) subgenus than with species of *L*. (*Leishmania*). Almost all markers were able to show correct size amplification products when tested with isolates of *L. guyanensis*, *L. braziliensis*, *L. peruviana* and *L. lainsoni* (Additional file 2: Table S5). Several markers did not amplify in some of the species evaluated, indicating potential null alleles.

### Genetic polymorphism and preliminary population data

**Fig. 1:**
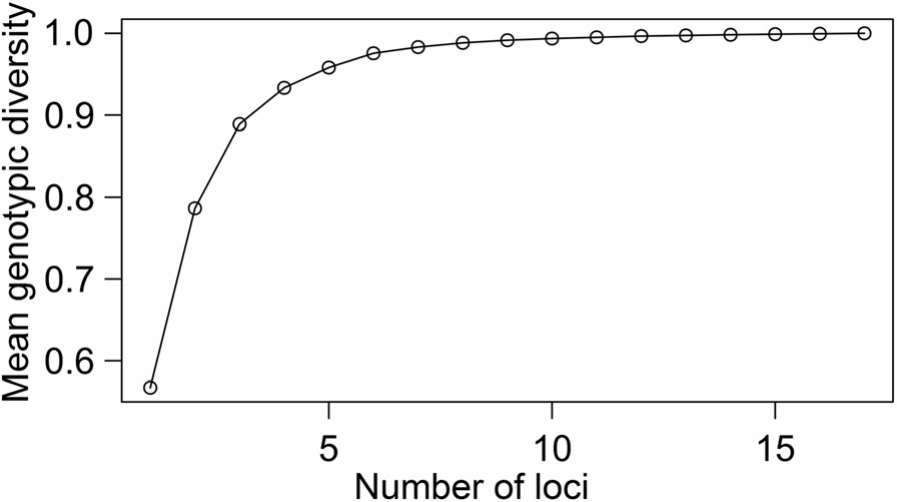
Plot of mean genotypic diversity as a function of the number of loci

Even when all *L. panamensis* isolates where collected from patients from a small geographic area, no repeated multilocus genotypes were detected (Additional file 2: Table S4). This result is congruent with AFLP data obtained with the same group of isolates, ruling out the hypothesis of full clonality in this population [3]. The polymorphism information content ranged from 0.071 (M75) to 0.877 (M233), with a mean value of 0.420 (Table 2). Markers M202 and M207 did not produce amplification products in isolates P7 and P14 respectively, indicating possible null alleles (Additional file 2: Table S4). Plotting genotypic diversity as a function of the number of loci showed that about 8–10 markers were enough to explain most of the genetic diversity in our sample of *L. panamensis* isolates (Fig. 1).

Observed heterozygosity (Ho) ranged from 0 to 0.556 with a mean value of 0.181 and was notably lower than the expected heterozygosity (He) for almost all loci (Table 2).

Within-population inbreeding coefficient (*F*_IS_) was first estimated by the method of moments, showing that all loci had positive *F*_IS_ (range 0.371-1; overall *F*_IS_ = 0.620, 95 % CI = 0.418-0.717), except markers M78 and M149. As an important step in the validation of the new microsatellite loci for population analyses, the possible presence of null alleles should be evaluated, particularly if excess homozygosis is observed. Several methods and algorithms have been reported in the literature to test microsatellites for possible null alleles [12]. However, most methods commonly used for that purpose make the assumption of Hardy-Weinberg equilibrium, which we cannot fulfil in the case of *Leishmania* data. Therefore, in order to verify if the observed positive value of *F*_IS_ was influenced by the possibility of having null alleles, we used Bayesian estimation of this coefficient and compared models considering or not the presence of null alleles in our data. This analysis revealed a large significant positive value (*F*_IS_ = 0.613, 95 % CI: 0.549-0.667). The model considering inbreeding (nfb) had a lower deviance information criterion (DIC = 1403) than the model considering only null alleles (nb, DIC = 1478), suggesting that inbreeding is the significant component of the model, rather than null alleles.

The exact test for Hardy-Weinberg equilibrium revealed a systematic departure from these theoretical proportions, as 13 out of 17 loci had significant *P* values (Table 2). Similarly, pairwise linkage disequilibrium (LD) was tested on all possible pairs of loci, showing significant values. Out of 136 comparisons, and expecting only seven significant values by chance, 40 pairs of loci showed *P* values below the strict Bonferroni corrected threshold (0.00036) (Additional file 2: Table S6).

**Fig. 2:**
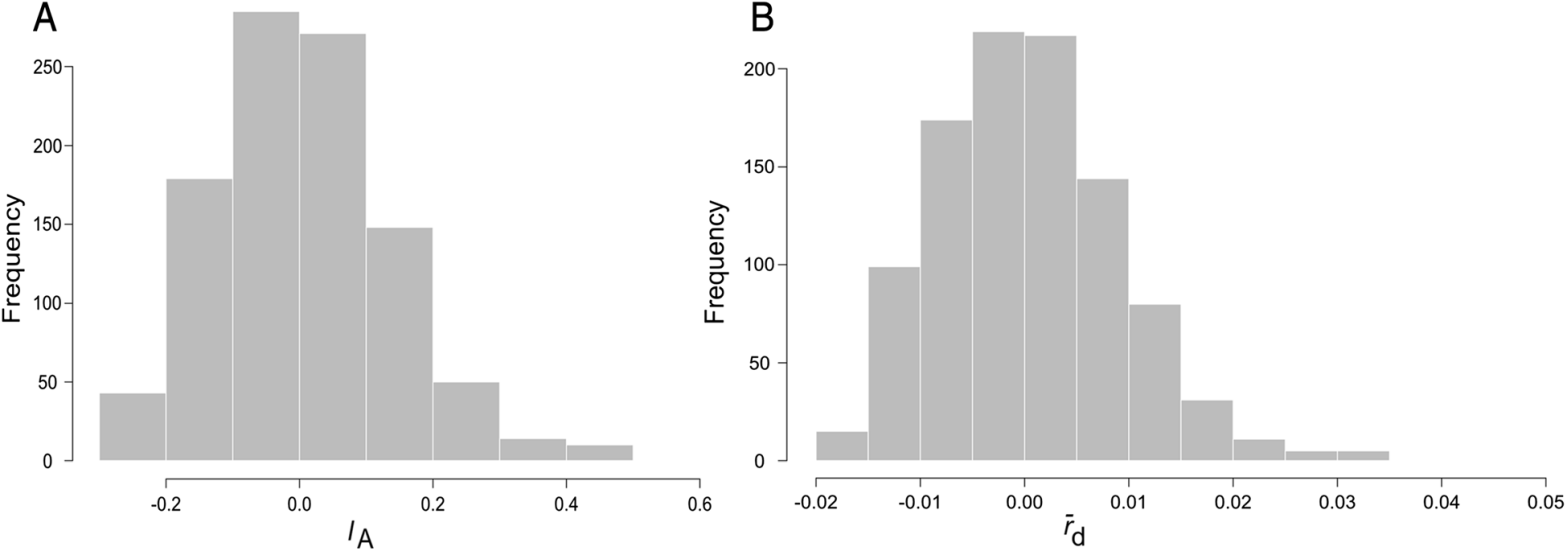
Analysis of multilocus linkage disequilibrium in *L. panamensis* microsatellite data. Bars represent the distribution of the statistics obtained from randomized datasets simulating free recombination. **a** resampling test for Index of Association (*I*_A_); **b** resampling test for $\overline{r}$_*d*_. The observed statistics in the original dataset were *I*_A_ = 2.19, $\overline{r}$_*d*_ = 0.145 (both *P* < 0.001)

**Fig. 3:**
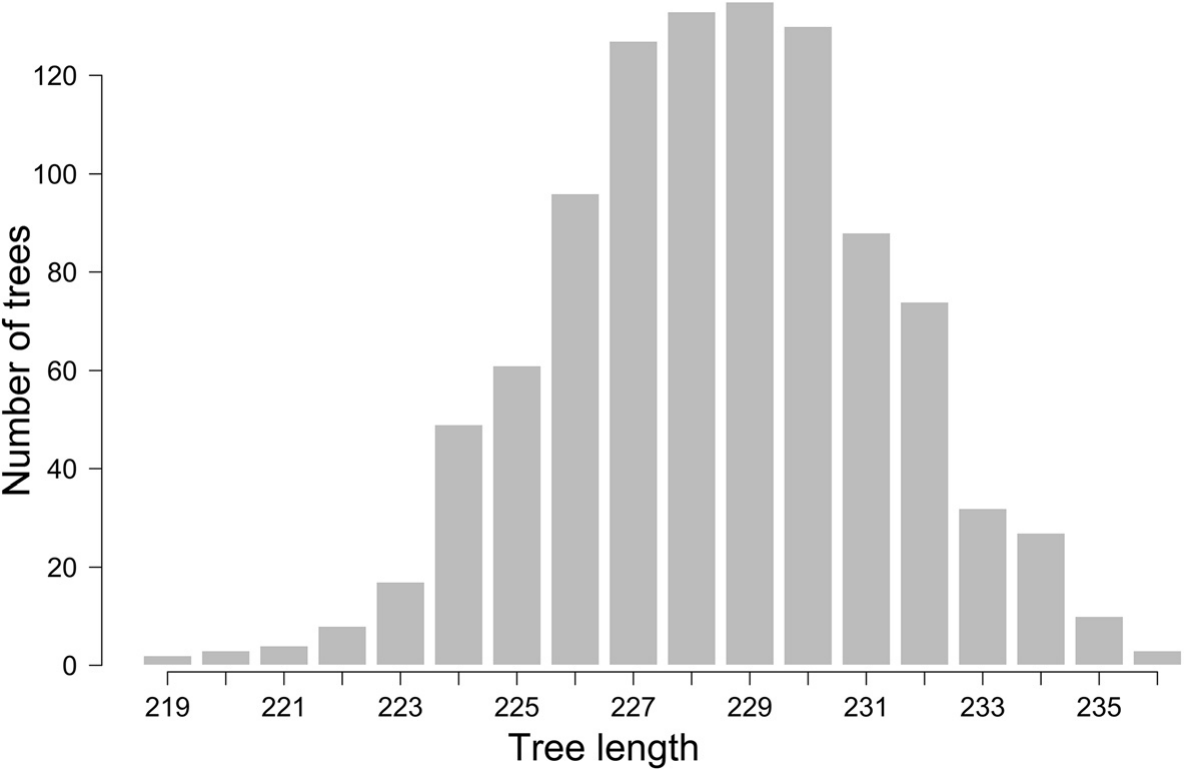
Analysis of recombination signature using parsimony tree length permutation test. Bars show the simulated distribution of tree lengths under the assumption of frequent recombination. Observed tree length = 163, *P* < 0.001

Furthermore, multilocus LD was assessed using the new microsatellites on our sample. While the expected mean values for measures of multilocus LD under panmixia are around cero, the values observed were significantly larger (*I*_A_ = 2.19 and $\overline{r}$_*d*_ = 0.145; both significant at *P* < 0.001) (Fig. 2a and b). The phylogenetic test for recombination shows an observed parsimony tree length of 163, significantly shorter (*P* < 0.001) than those generated from shuffled datasets (mean tree length: 229 steps, Fig. 3). Both approaches allow rejection of the null hypothesis of panmixia. Using a different marker system on the same group of isolates, we have already shown strong signatures of LD, as independent AFLP markers generated highly correlated distance matrices between isolates [3]. Diverse levels of LD have been reported in various *Leishmania* species using several marker systems [13–15]. Due to the limited sample size we cannot rule out that some additional factors may be influencing the LD estimations reported here, such as Wahlund effect, hidden population subdivision, cryptic species within the sampling units, recombination rate, genetic drift, mutation rates, epistasis or selection.

Although several tests performed on our data allow rejection of the hypothesis of panmixia/frequent recombination, the values of association indexes as well as those of inbreeding coefficient are not consistent with strict clonality either. Predominant clonality would produce strongly negative *F*_IS_ values and a higher index of association (*I*_A_ and $\overline{r}$_*d*_). Taken together, these results suggest a mixed mode of reproduction that involves both clonality and selfing with sporadic recombination. The same mode of reproduction has been suggested for other species of the *L*. (*Viannia*) subgenus, including *L. braziliensis* and *L. guyanensis* [13, 16] as well as some other parasites such as *Trypanosoma brucei* and *Plasmodium falciparum* [17, 18]. In the closely related *L. guyanensis*, a simulation approach has shown that a reproductive mating scheme involving frequent sexual events may account for the observed levels of linkage disequilibrium and heterozygote deficit [16].

We have developed and tested a new panel of microsatellites using *L. panamensis* genomic sequence data. The bioinformatic pipeline employed allowed us to identify significantly more potential loci for evaluation than previously reported in *Leishmania* using conventional methodologies [19, 20]. The new panel of microsatellites will contribute to the existing tools for better understanding of the distribution and dynamics of the genetic diversity in this important parasite.

## Additional files

Additional file 1:
**Extended Methods.** (DOCX 32 kb) Additional file 2: Supplementary Data
**Table S1. ** Perfect microsatellite loci found in *L. panamensis* genome. **Table S2.** Specificity of new *L. panamensis* SSR panel on non-target DNAs. **Table S3.** Characterization of limits of detection for each described *L. panamensis* SSR locus. **Table S4.** Raw genotype data for all tested loci and *Leishmania* species. **Table S5.** Amplification profiles of described panel of microsatellites in species other than *L. panamensis*. **Table S6.** *P* values for exact test of pairwise linkage disequilibrium, as estimated in Arlequin v3.5.1.3. (XLSX 28 kb)
