# Supplementary material for: Genome-wide discovery and development of polymorphic microsatellites from Leishmania panamensis parasites circulating in central Panama
Source: Parasit Vectors. 2015 Oct 12;8:527. doi: 10.1186/s13071-015-1153-2 (PMC4603350; doi:10.1186/s13071-015-1153-2)
Supplement: Additional file 1: — Extended Methods. (DOCX 32 kb) [file 13071_2015_1153_MOESM1_ESM.docx]

# Genome-wide discovery and development of polymorphic microsatellites from *Leishmania panamensis* parasites circulating in central Panama

# Extended Methods

## Ethics Statement

Approval for this research was obtained from the INDICASAT AIP institutional review board. The parasite isolates were treated blindly with no relation to the source patients. At the moment of isolation, written informed consents were obtained from patients. Data was analysed anonymously.

## Parasite culture and DNA isolation

The isolates used for this work had been obtained for diagnostic purposes and already described in previously published research [1]. *Leishmania* isolates were recovered from cutaneous leishmaniasis patients in Panama province using standard biopsy procedures. Reference strains for different species were obtained from several institutions, including cryobanks at University of Panama, Walter Reed Army Institute of Research, Instituto Conmemorativo Gorgas de Estudios de la Salud, the *Leishmania* collection at the Instituto Osvaldo Cruz (CLIOC) and INDICASAT AIP. Primary parasite isolations were done in NNN biphasic medium [2] at room temperature. For DNA extraction promastigotes were cultured in T25 tissue culture flasks containing 10 ml of Schneider’s insect medium (Sigma, USA) plus gentamicin (50 µg/ml) and 20% (v/v) heat-inactivated fetal calf serum at 25°C. Reference strain PSC-1 (MHOM/PA/94/PSC-1) was cloned following the hanging drop procedure [3]. Total genomic DNA was extracted from promastigote cultures using standard procedures (Wizard Genomic DNA purification kit, Promega, USA). The DNA concentration and quality were analysed with a NanoDrop 2000 spectrometer (Thermo Scientific, USA). Species identities were verified by *hsp70* gene PCR-RFLP using HaeIII and BccI, MluI or RsaI restriction endonucleases.

## High-throughput screening for microsatellite loci

We used the genome of *L*. *panamensis* strain PSC-1 for high-throughput microsatellite screening [4]. This genome was sequenced and assembled by our group, and it has been deposited in Genbank under bioproject PRJNA235344, with accession numbers CP009370 to CP009404 for chromosomes 1 to 35, respectively. MSATCOMMANDER v1.08 [5] was used to screen the chromosome sequences for microsatellite motifs. Only perfect di-, tri-, tetra-, and pentanucleotide motifs were considered, all with at least five repeated units. PCR amplification primers were designed for each detected loci using the MSATCOMMANDER built-in wrapper to Primer3 [6] using the default options.

Candidates for synthesis were selected by using several Perl scripts developed in-house. We considered primer pairs with only one predicted amplification product in *Leishmania*. Search of possible amplification products for each primer pair was performed by using the blastn program from the BLAST suite (v2.2.25+) [7]. A query sequence was prepared for each primer pair by concatenating the sequence of the upper primer to the reversed complementary sequence of the corresponding lower primer, both separated by a stretch of 10 undefined positions (“N”). To improve the sensitivity while searching with such query sequences, the word size (“–word_size” flag of blastn) was changed to 7 (the minimum allowed), and the threshold for the E-value was chosen to be relatively large (< 10). When using query sequences prepared this way, BLAST attempts to locate the sequence of the upper and/or lower primer in the target sequence, regardless if they are contiguous or separated by an arbitrary number of bases. Potential amplification products were then predicted when high scoring pairs matching the upper and lower primers were found, but separated by a distance within the range of the expected amplification product size (up to 500 bp).

The strategy described above was used to search for possible products in our *L*. *panamensis* reference genome and in the genome sequences of five *Leishmania* species; namely *L*. *major* [8], *L. infantum* [9], *L. braziliensis* [9], *L. donovani* [10] and *L. mexicana* [11]. Known sequences for kinetoplast DNA (kDNA) were also screened to help distinguish kDNA markers.

While all tetra- and pentanucleotide loci initially selected were considered for testing, di- and trinucleotides were additionally filtered to fit in the range of 10-17 and 7-21 repeats respectively. The latter was done to increase the probability to identify polymorphic loci while maintaining low stuttering levels. After the bioinformatic filtering, 406 microsatellite loci including di-, tri-, tetra- and pentanucleotide motifs were selected for primer synthesis and subsequent testing of polymorphisms.

## Polymorphic microsatellite marker identification, optimization and evaluation

A three-primer PCR method was designed for simplex analyses by incorporating a universal tag (“Tail A”) to the 5´ end of forward primers [12]. Briefly, PCR reactions were performed in a total volume of 25 µl containing 1 ng of genomic DNA, 1X PCR Master Mix (Promega, USA), 0.2 µM fluorescently labelled universal primer, 0.15 µM forward tailed primer and 0.5 µM reverse primer. PCR was carried out using an initial denaturation at 94 °C (2 min), 30 cycles consisting of denaturation at 94 °C (30 seconds), annealing at 56 °C (30 seconds), and extension at 72 °C (1 min) and a final extension at 72 °C (30 min) with a 2720 Thermal Cycler (Applied Biosystems, USA). Microsatellite alleles were detected on a Genetic Analyzer 3130 using GeneScan ROX 500 as internal size standard (Applied Biosystems, USA). Electropherograms were analysed using GeneMarker v2.20 (SoftGenetics LLC, USA) using the default settings for fragment analysis, but adjusting the stutter peak filter percentage to 90% (left) and 30% (right) and the peak detection threshold at a minimum of 100 RFU.

Screening for polymorphisms was done following additional steps. First, amplification reactions were done using two pools of DNA, each one combining equal amounts of genomic DNA from ten isolates. Then, after elimination of non-amplifying, non-polymorphic and high stutter loci, PCR reactions were repeated with DNA from individual isolates to confirm polymorphisms. Additional annealing temperature optimization was performed for the selected 17 polymorphic loci by assessing amplification products from PCR reactions varying in 2 °C increments.

Specificity and sensitivity tests were performed using the optimized PCR conditions for each locus. Specificity was determined performing PCR amplifications with non-related DNA from *Lutzomyia* sp. and *Homo sapiens*, and also with DNA from several *Leishmania* species from the two subgenera. The species tested included *L. mexicana*, *L. major*, *L. aristidesi*, *L. donovani*, *L. infantum*/*chagasi*, *L. guyanensis*, *L. braziliensis*, *L. peruviana*, and *L. lainsoni*. Sensitivity of the assays for detection of each locus was performed using variable amounts of *L. panamensis* DNA, ranging from 1 to 0.01 ng per reaction.

## Data analysis

The genetic polymorphism of the microsatellite loci was evaluated by number of genotypes, number of alleles, observed heterozygosity, expected heterozygosity, and polymorphism information content using POWERMARKER v3.25 [13]. Exact tests for Hardy-Weinberg equilibrium and pairwise linkage disequilibrium (LD) were done as implemented in Arlequin v3.5.1.3 [14]. Hardy-Weinberg test was done using a Markov chain length of 1 000 000 and 100 000 dememorization steps. For pairwise LD the run employed 20 000 permutations and 5 initial conditions for EM algorithm. A plot analysis of genotypic diversity as a function of the number of loci was performed using Multilocus v1.3b to check whether scoring more loci was required to describe the genotypic diversity of our sample [15]. The within-population inbreeding coefficient *F*_IS_ [16] was estimated using the method of moments [17] as implemented in POWERMARKER . The 95% confidence interval was estimated by bootstrapping over loci using 10 000 permutations.

It is known that null alleles can artificially inflate the proportion of homozygotes. To check to what extent null alleles played a role, we used the software INEST v2.0 [18] to perform Bayesian estimation of unbiased multilocus inbreeding coefficient using 200 000 cycles, 1000 thinning and burn-in set to 20 000. The software evaluates two models through Deviance Information Criterion (DIC) values to assess the significance of inbreeding and null alleles.

Associations among SSR loci were tested by estimating the Index of association (*I*_A_) and the related modified statistic $\bar{r}$*_d_* as implemented in Multilocus. *I*_A_ is low if recombination is occurring and high if clonal structure is predominant. As *I*_A_ is affected by loci number, the derived index $\bar{r}$*_d_* estimates the same parameter adjusted to the range 0 to 1 and is independent of loci number. Statistical significance is tested by randomization of alleles among isolates.

To further interrogate SSR data for signatures of clonality or recombination, a parsimony tree length permutation test was performed as implemented in PAUP* v4.0b10 (PTP option, 1000 permutations) [19]. The allele size coded data was converted to a presence - absence matrix and analysed using parsimony criterion considering isolates as taxa. This test compare the observed parsimony tree length with the distribution of tree lengths obtained from permuted datasets, assuming that clonality would induce a significantly shorter tree in the observed data set [20-22]. Deviation from the distribution of permuted tree lengths allows rejection of the null hypothesis of panmixia.

# References

1. Restrepo CM, De La Guardia C, Sousa OE, Calzada JE, Fernández PL, Lleonart R: **AFLP polymorphisms allow high resolution genetic analysis of American Tegumentary Leishmaniasis agents circulating in Panama and other members of the *Leishmania* genus.** *PLoS One* 2013, **8**:e73177.

2. Nicole C: **Culture du parasite du Bouton’Orient.** *C R Acad Sci* 1908, **146**:842–843.

3. Evans DA, Smith V: **A simple method for cloning leishmanial promastigotes.** *Z Parasitenkd* 1986, **72**:573–6.

4. Llanes A, Restrepo CM, Del Vecchio G, Anguizola FJ, Lleonart R: **The genome of *Leishmania panamensis*: insights into genomics of the *L*. (*Viannia*) subgenus**. *Sci Rep.* 2015:8550. doi: 10.1038/srep08550.

5. Faircloth BC: **msatcommander: detection of microsatellite repeat arrays and automated, locus-specific primer design.** *Mol Ecol Resour* 2008, **8**:92–4.

6. Rozen S, Skaletsky H: **Primer3 on the WWW for general users and for biologist programmers.** *Methods Mol Biol* 2000, **132**:365–86.

7. Altschul SF, Gish W, Miller W, Myers EW, Lipman DJ: **Basic local alignment search tool.** *J Mol Biol* 1990, **215**:403–10.

8. Ivens AC, Peacock CS, Worthey EA, Murphy L, Aggarwal G, Berriman M, Sisk E, Rajandream M-A, Adlem E, Aert R, Anupama A, Apostolou Z, Attipoe P, Bason N, Bauser C, Beck A, Beverley SM, Bianchettin G, Borzym K, Bothe G, Bruschi C V, Collins M, Cadag E, Ciarloni L, Clayton C, Coulson RMR, Cronin A, Cruz AK, Davies RM, De Gaudenzi J, et al.: **The genome of the kinetoplastid parasite, *Leishmania major*.** *Science* 2005, **309**:436–42.

9. Peacock CS, Seeger K, Harris D, Murphy L, Ruiz JC, Quail M a, Peters N, Adlem E, Tivey A, Aslett M, Kerhornou A, Ivens A, Fraser A, Rajandream M-A, Carver T, Norbertczak H, Chillingworth T, Hance Z, Jagels K, Moule S, Ormond D, Rutter S, Squares R, Whitehead S, Rabbinowitsch E, Arrowsmith C, White B, Thurston S, Bringaud F, Baldauf SL, et al.: **Comparative genomic analysis of three *Leishmania* species that cause diverse human disease.** *Nat Genet* 2007, **39**:839–47.

10. Downing T, Imamura H, Decuypere S, Clark TG, Coombs GH, Cotton JA, Hilley JD, de Doncker S, Maes I, Mottram JC, Quail MA, Rijal S, Sanders M, Schönian G, Stark O, Sundar S, Vanaerschot M, Hertz-Fowler C, Dujardin JC, Berriman M: **Whole genome sequencing of multiple *Leishmania donovani* clinical isolates provides insights into population structure and mechanisms of drug resistance.** *Genome Res* 2011, **21**:2143–56.

11. Rogers MB, Hilley JD, Dickens NJ, Wilkes J, Bates PA, Depledge DP, Harris D, Her Y, Herzyk P, Imamura H, Otto TD, Sanders M, Seeger K, Dujardin JC, Berriman M, Smith DF, Hertz-Fowler C, Mottram JC: **Chromosome and gene copy number variation allow major structural change between species and strains of *Leishmania*.** *Genome Res* 2011, **21**:2129–42.

12. Blacket MJ, Robin C, Good RT, Lee SF, Miller AD: **Universal primers for fluorescent labelling of PCR fragments--an efficient and cost-effective approach to genotyping by fluorescence.** *Mol Ecol Resour* 2012, **12**:456–63.

13. Liu K, Muse S V: **PowerMarker: an integrated analysis environment for genetic marker analysis.** *Bioinformatics* 2005, **21**:2128–9.

14. Excoffier L, Lischer HEL: **Arlequin suite ver 3.5: a new series of programs to perform population genetics analyses under Linux and Windows.** *Mol Ecol Resour* 2010, **10**:564–7.

15. Agapow P, Burt A: **Indices of multilocus linkage disequilibrium**. *Mol Ecol Notes* 2001, **1**:101–102.

16. Wright S: **The interpretation of population structure by F-statistics with special regard to systems of mating.** *Evolution (N Y)* 1965, **19**:395–420.

17. Weir B: *Genetic Data Analysis II.* Sunderland, MA: Sinauer Associates, Inc; 1996.

18. Chybicki IJ, Burczyk J: **Simultaneous estimation of null alleles and inbreeding coefficients.** *J Hered* 2009, **100**:106–13.

19. Swofford D: **PAUP*: Phylogenetic Analysis Using Parsimony.** 2002.

20. Burt A, Carter DA, Koenig GL, White TJ, Taylor JW: **Molecular markers reveal cryptic sex in the human pathogen *Coccidioides immitis*.** *Proc Natl Acad Sci U S A* 1996, **93**:770–3.

21. Carter DA, Burt A, Taylor JW, Koenig GL, White TJ: **Clinical isolates of *Histoplasma capsulatum* from Indianapolis, Indiana, have a recombining population structure.** *J Clin Microbiol* 1996, **34**:2577–84.

22. Halliday CL, Carter DA: **Clonal reproduction and limited dispersal in an environmental population of *Cryptococcus neoformans* var *gattii* isolates from Australia.** *J Clin Microbiol* 2003, **41**:703–11.
